# Macroscopic Entropy of Non-Equilibrium Systems and Postulates of Extended Thermodynamics: Application to Transport Phenomena and Chemical Reactions in Nanoparticles

**DOI:** 10.3390/e20100802

**Published:** 2018-10-18

**Authors:** Sergey I. Serdyukov

**Affiliations:** 1 Department of Chemistry, Moscow State University, 119992 Moscow, Russia; babkin-msu@yandex.ru; Tel.: +7-(919)-964-09-32; 2 Topchiev Institute of Petrochemical Synthesis, Russian Academy of Sciences, 119991 Moscow, Russia

**Keywords:** extended irreversible thermodynamics, thermodynamic postulates, linear irreversible thermodynamics, nanosystems, non-classical transport phenomena, diffusion and chemical reactions in nanosystems

## Abstract

In this work, we consider extended irreversible thermodynamics in assuming that the entropy density is a function of both common thermodynamic variables and their higher-order time derivatives. An expression for entropy production, and the linear phenomenological equations describing diffusion and chemical reactions, are found in the context of this approach. Solutions of the sets of linear equations with respect to fluxes and their higher-order time derivatives allow the coefficients of diffusion and reaction rate constants to be established as functions of size of the nanosystems in which these reactions occur. The Maxwell-Cattaneo and Jeffreys constitutive equations, as well as the higher-order constitutive equations, which describe the processes in reaction-diffusion systems, are obtained.

## 1. Introduction

Actually, irreversible processes which occur in micro and nanostructures attract more attention of researchers. Extensive development of nanotechologies and creation and construction of devices at micro- and nanoscales has promoted numerous theoretical studies in this field of science [1,2]. One of the most promising routes to study properties of nanosystems and multilayer systems is non-equilibrium thermodynamics. Classical irreversible thermodynamics is based on the local-equilibrium hypothesis, which implies equilibrium in each small volume of the system, although the non-uniform system as a whole remains in non-equilibrium [3,4,5]. Due to the fact that in a locally equilibrium system it is possible to have gradients of state variables, the entropy density in each small volume depends on the internal energy density per unit volume and on the partial densities of the medium components. Such an approach enables to pass from thermodynamic description of equilibrium systems to non-equilibrium ones and to formulate the main theses of irreversible thermodynamics, such as the theorem of minimum entropy production and the general evolutionary criterion [6,7]. 

If we meanwhile consider the very fast or very abrupt processes, then many properties of non-equilibrium systems cannot be described in the context of local-equilibrium approximation. The validity of local equilibrium is determined by criteria associated to internal microscales of the medium and to external macroscales [8,9,10]. Microscales of a medium are the characteristic scales of the medium microstructure and characteristic relaxation times of dissipative fluxes, which are determined by the parameters of atoms and molecules (e.g., for gases they are mean free path and mean time between two collisions of molecules, respectively). Macroscales is the characteristic macroscale of the system and characteristic time of changing of macroscopic parameters. In our case, spatial macroscale is a linear dimension of a system whether it is a particle size or a nanolayer thickness. When a spatial macroscale is much larger than the relevant microscale (at constant time scales), then local-equilibrium hypothesis remains valid that enables us to use classical irreversible thermodynamics. In turn, if a spatial macroscale is comparable or smaller than the microscale, there is no local equilibrium and the non-equilibrium system has to be described in the contents of more general theory. 

The latest development in irreversible thermodynamics led to elaboration of extended irreversible thermodynamics (EIT) which allowed study of fast and steep phenomena [11,12,13,14,15]. Extended irreversible thermodynamics involves investigations of irreversible processes with high frequencies and small length scales, which take place in nanosystems [16]. Heat conduction in thin layers [1,2], diffusion and chemical reactions in nanoparticles [17], as well as nanocatalysis [18,19] are of great interest in the context of EIT, where memory and non-local effects are taken into consideration [20]. 

Extended irreversible thermodynamics is based on the generalization of classical local-equilibrium theory by using additional thermodynamic variables (or extra variables). Their choice is an important problem which can be solved by different ways. The most common route to extend a classical theory is the introduction of dissipative fluxes as extra variables [11,12,13,14]. For this kind of EIT, variables are both well-known fluxes, which arise in balance equations, and higher-order fluxes (fluxes of the fluxes) that allows the unlimited number of extra variables. 

Another type of EIT involves the time derivatives of common thermodynamic variables as extra variables [21,22,23,24,25]. In this case, the local-equilibrium hypothesis is not applicable, and we must proceed from a less strict postulate. Such a hypothesis is the local uniformity hypothesis [26]. It implies that the system which is non-uniform as a whole will be considered uniform at each point. Then the entropy density depends on the higher-order time derivatives of the density of the internal energy and the partial densities. Based on this postulate, one can construct the thermodynamic formalism which is similar to classical, where the main laws and consequences of non-equilibrium thermodynamics are preserved [27]. In this paper, we have used for the first time the approach based on the works [21,22,23,24,25,26,27] for describing the properties of nanosystems.

The process of thermal conduction has been widely investigated, and we turn our attention to the reaction-diffusion processes. Our theory will be based on the linear extended thermodynamics which avoids using the Fourier’s transform and some additional simplifications. Therefore, we hope to obtain a new higher-order constitutive equations. The linear theory will lead to Equations (63) and (64), which describe the dependence of the diffusion coefficient and the rate of chemical reaction on the size of nanoparticles. Apart from this, we will introduce a new definition of the generalized temperature and chemical potentials, which will allow extension of a class of constitutive equations, the Jeffreys type equations included.

In Section 2, we will focus on heat transport and reaction-diffusion processes in the context of classical irreversible thermodynamics that is based on the local-equilibrium hypothesis. Section 3 is devoted to a study of the properties of nanosystems, using the traditional EIT, where the extra variables are higher-order fluxes of heat flux. The atypical version of EIT will be considered in Section 4, where we will characterize non-equilibrium systems relying on a postulate in which the additional variables are the higher-order time derivatives of partial densities of the components. Section 5 is dedicated to two-component systems, where we will consider the appropriate constitutive equations. In particular, the diffusion processes and chemical reaction occurring in nanosystems will be studied. As a result, using linear systems of phenomenological equations, we will obtain for the first time Equations (63) and (64) describing the dependence of the diffusion coefficient and the rate of chemical reaction on the size of nanoparticles. The characteristic parameters—relaxation times, spatial characteristic magnitudes and relevant characteristic rates—will be discussed in Section 6. In Section 7 and Section 8, the multi-dimensional case of phenomenological equations and the appropriate higher-order constitutive equations, which describe the reaction-diffusion processes in nanosystems, will be considered.

## 2. Classical Irreversible Thermodynamics of Heat Conduction, Diffusion and Chemical Reactions

Classical irreversible thermodynamics (CIT) [3,4,5,6,7] is based on the local-equilibrium hypothesis, which leads to the fundamental equation in the local form:

(1) $$T\partial_{t}s=\partial_{t}u-\underset{i}{\sum }\mu_{i}\partial_{t}\rho_{i}$$

where *T* is the local-equilibrium temperature, $\mu_{i}$ are the chemical potential i=1, 2, …,K; u is the density of the internal energy per unit volume, s is the density of entropy per unit volume, $\rho_{i}$ is the partial density of the *i*-th component (i=1, 2, 3,…,K, at that all components are assumed to be independent [28]), *t* is the time. 

First, we will consider the classical heat conduction in rigid bodies, which is the simplest thermodynamics theory. Then Equation (1) can be simplified as:

(2) $$T\partial_{t}s=\partial_{t}u$$

that means the entropy density is a function of the internal energy: s=s(u). The balance equation that connects u with the heat flux q is being written, as follows:

(3) $$\partial_{t}u=-\nabla \cdot q$$

The fundamental Equation (2) and the balance Equation (3) lead to the standard entropy balance equation (i.e., in the form $\partial_{t}s=-\nabla \cdot J_{s}+\sigma_{s}$):

(4) $$\partial_{t}s=-\nabla \cdot \left(T^{-1}q\right)+q\cdot \nabla T^{-1}$$

The expression for the entropy flux $J_{s}=T^{-1}q$ and the entropy production results from Equation (4):

(5) $$\sigma_{s}=q\cdot \nabla T^{-1}\geq 0$$

where a sign is defined by the second law of thermodynamics. The linear relationship between the flux q and the thermodynamic force $\nabla T^{-1}$ results in one phenomenological equation:

(6) $$\nabla T^{-1}=R_{q}q$$

which ensures the positivity of entropy production (5) at $R_{q}>0$. The phenomenological Equation (6) allows the heat flux to be expressed as:

(7) $$q=-\frac{1}{R_{q}T^{2}}\nabla T$$

Relation (7), by comparing it with the Fourier’s law:

$$q=-\lambda_{q}\nabla T$$

defines the coefficient $R_{q}$:

(8) $$R_{q}=\frac{1}{\lambda_{q}T^{2}}$$

Thus, introducing the linear relationship between the flux and the force, we have adapted the Fourier’s law for heat conduction that guaranties the positiveness of entropy production within the framework of CIT. 

Next we will consider only dissipative processes [6] at constant density of internal energy *u* ($\partial_{t}u=0$) in the absence of convective motion (diffusion and chemical reactions at v=0 and dv/dt=0, where v is the velocity of the multicomponent medium). The fundamental equation in the local form (1) is therefore reduced to the following:

(9) $$\partial_{t}s=-\underset{i}{\sum }T^{-1}\mu_{i}\partial_{t}\rho_{i}$$

As is seen in Equation (9), the entropy density in the considered case is a function of partial densities of the independent components:

(10) $$s=s\left(\rho_{1},\rho_{2},\ldots ,\rho_{K}\right)$$

Any changes in partial densities over time $\partial \rho_{i}/\partial t$ obey the balance equations [4,5]

(11) $$\partial_{t}\rho_{i}=-\nabla \cdot J_{i}+\sum_{r}\nu_{ir}w_{r},\;i=1,2,\ldots ,K$$

where $J_{i}$ is the diffusion flux of the *i*-th component, $w_{r}$ is the velocity of a chemical reaction *r* (r=1,2,…,Q), $\nu_{ir}$ is the stoichiometric coefficient of the *i-*th component in the reaction r. In the present work we will pay attention to the following chemical reactions:

(12) $$\nu_{1r}X_{1}+\nu_{2r}X_{2}+\ldots \nu_{Lr}X_{L}=\nu_{\left(L+1\right)r}Y_{L+1}+\nu_{\left(L+2\right)r}Y_{L+2}+\ldots +\nu_{Kr}Y_{K},\;r=1,2,\ldots ,Q$$

The fundamental Equation (9) and the balance Equation (11) define the formalism of classical non-equilibrium thermodynamics. Using Equation (11), we substitute $\partial \rho_{i}/\partial t$ in Equation (9). After some transformations, we have the entropy balance equation:

(13) $$\partial_{t}s=-\nabla \cdot \left(\underset{i}{\sum }T^{-1}\mu_{i}J_{i}\right)+\underset{i}{\sum }J_{i}\cdot \nabla \left(T^{-1}\mu_{i}\right)+\underset{r}{\sum }T^{-1}w_{r}A_{r}$$

where $A_{r}=-\sum_{i}\nu_{ir}\mu_{i}$ is the affinity of chemical reaction in Equation (12) (stoichiometric coefficients for the initial components are taken with a negative sign, and those for products are denoted with a positive sign). The balance Equation (13) is written in a standard form, resulting in the entropy flux $J_{s}=\sum_{i}T^{-1}\mu_{i}J_{i}$ and entropy production:

(14) $$\sigma_{s}=\underset{i}{\sum }J_{i}\cdot \nabla \left(T^{-1}\mu_{i}\right)+\underset{r}{\sum }T^{-1}w_{r}A_{r}\geq 0$$

The inequality sign in Equation (14) (positivity of entropy production) is determined by the second law of thermodynamics. Entropy production has a bilinear form, where the vector fluxes $J_{i}$ are multiplied by the appropriate vector forces $\nabla \left(T^{-1}\mu_{i}\right)$, and the scalar fluxes $w_{r}$ are multiplied by the scalar forces $A_{r}/T$. According to the Curie principle, there is no interaction between the diffusion fluxes and with the velocity of chemical reaction. Then each term in (14) is non-negative:

(15) $$\sigma_{s}^{\mathrm{diff}}=J_{i}\cdot \nabla \left(T^{-1}\mu_{i}\right)\geq 0,\;i=1,2,\ldots ,K$$

(16) $$\sigma_{s}^{\mathrm{react}}=T^{-1}w_{r}A_{r}\geq 0,\;r=1,2,\ldots ,Q$$

According to the Equations (15) and (16), each of fluxes $J_{1},J_{2},\ldots ,J_{K}$ and $w_{1},w_{2},\ldots ,w_{Q}$ depends only on the corresponding thermodynamic force $\nabla \left(T^{-1}\mu_{1}\right),\nabla \left(T^{-1}\mu_{2}\right),\ldots ,\nabla \left(T^{-1}\mu_{K}\right)$ and $T^{-1}A_{1},T^{-1}A_{2},\ldots ,T^{-1}A_{Q}$, respectively. Further we will focus on the linear correlation between force and flux:

(17) $$\nabla \left(T^{-1}\mu_{i}\right)=R^{\left(i\right)}J_{i},\;i=1,2,\ldots ,K$$

(18) $$T^{-1}A_{r}=\lambda^{\left(r\right)}w_{r},\;r=1,2,\ldots ,Q$$

where $R^{\left(1\right)},R^{\left(2\right)},\ldots ,R^{\left(K\right)}$ and $\lambda^{\left(1\right)},\lambda^{\left(2\right)},\ldots ,\lambda^{\left(Q\right)}$ are the phenomenological coefficients. Expressing the flux through the thermodynamic force in Equation (17) results in a well-known Fick’s law. Assuming that the chemical potential $\mu_{i}$ depends on the component concentration $n_{i}$ ($=\rho_{i}/M_{i}$, where $M_{i}$ is the molar mass of the *i*-th component), we obtain $J_{i}=\frac{1}{R^{\left(i\right)}}\nabla \left(T^{-1}\mu_{i}\right)=\frac{1}{R^{\left(i\right)}}\frac{\partial \left(T^{-1}\mu_{i}\right)}{\partial n_{i}}\nabla n_{i}$. 

Denoting the coefficient before $\nabla n_{i}$ through $-D_{0i}$, we have Fick’s law:

(19) $$J_{i}=-D_{0i}\nabla n_{i},\;\;\;\;\;\;r=1,2,\ldots ,K$$

In accordance with Equation (19), the concentration gradient $\nabla n_{i}$ induces one diffusion flux $J_{i}$. We have therefore justified the Fick’s law in the context of classical non-equilibrium thermodynamics. 

The linear dependence of the velocity of chemical reaction on thermodynamic force is being found from Equation (18):

(20) $$w_{r}=\frac{1}{\lambda^{\left(r\right)}}\frac{A_{r}}{T},\;\;r=1,2,\ldots ,Q$$

On the other hand, the velocity of chemical reaction $w_{r}$ depends on the forward rate $w_{r}^{+}=k_{0r}^{+}n_{1}^{\nu_{1r}}n_{2}^{\nu_{2r}}\ldots n_{L}^{\nu_{Lr}}$ and the reverse rate $w_{r}^{-}=k_{0r}^{-}n_{L+1}^{\nu_{L+1}}n_{L+2}^{\nu_{L+2}}\ldots n_{K}^{\nu_{K}}$; i.e., $w_{r}=w_{r}^{+}-w_{r}^{-}$, where $k_{0r}^{+}$ is the constant of the forward rate, $k_{0r}^{-}$ is the constant of the reverse rate. Using the known expression for the chemical potential $\mu_{i}=\mu_{i}{}^{0}+ℜT\;ln\;n_{i}$, we obtain [7]:

(21) $$A_{r}=-\underset{i}{\sum }\nu_{ir}\mu_{i}=ℜT\;ln\frac{w_{r}^{+}}{w_{r}^{-}}$$

where ℜ is the universal gas constant. The relationship between the rates of forward and reverse reactions follows from Equation (21): $w_{r}^{-}=w_{r}^{+}\exp\left(-\frac{A_{r}}{ℜT}\right)$ and the velocity of chemical reaction in the linear approximation is:

(22) $$w_{r}=w_{r}^{+}-w_{r}^{-}=w_{r}^{+}\left[1-\exp\left(-\frac{A_{r}}{ℜT}\right)\right]\approx \frac{w_{r}^{+}}{ℜ}\frac{A_{r}}{T}$$

Comparing Equations (20) and (22), we have:

(23) $$\lambda^{\left(r\right)}=\frac{ℜ}{w_{r}^{+}}$$

Expressing $w_{r}^{+}$ through the component concentrations, the velocity of reaction is being established from Equation (22) in the form:

(24) $$w_{r}=k_{0r}^{+}n_{1}^{\nu_{1r}}n_{2}^{\nu_{2r}}\ldots n_{L}^{\nu_{Lr}}\frac{A_{r}}{ℜT}$$

The analogous equality can be obtained by expressing the velocity of reaction through $w_{r}^{-}$. 

The classical thermodynamic theory will be expanded below for both the heat conduction and diffusion and chemical reaction, using in the context various initial postulates of extended irreversible thermodynamics. 

## 3. Traditional Version of EIT and Higher-Order Heat Fluxes 

Extended non-equilibrium thermodynamics is a generalization of classical one by introducing extra variables [11]. In this section we will consider the approach in the context of EIT, which implies that the extra variables are the higher-order fluxes (fluxes of the fluxes):

(25) $$q^{\left(1\right)},q^{\left(2\right)},\ldots ,q^{\left(N\right)}$$

where $q^{\left(1\right)}=q$, $q^{\left(2\right)}$ is the flux of the flux $q^{\left(1\right)}$ (second-order tensor), $q^{\left(3\right)}$ is the flux of $q^{\left(2\right)}$, and so on. Higher-order fluxes (Equation (25)) satisfy a set of the balance equations:

(26) $$\begin{array}{c} \partial_{t}q=-\nabla \cdot q^{\left(2\right)}+\sigma^{\left(2\right)}\\ \partial_{t}q^{\left(2\right)}=-\nabla \cdot q^{\left(3\right)}+\sigma^{\left(3\right)}\\ \begin{array}{c} .........................................\\ \partial_{t}q^{\left(N\right)}=-\nabla \cdot q^{\left(N+1\right)}+\sigma^{\left(N\right)} \end{array} \end{array}$$

where $\sigma^{\left(1\right)},\sigma^{\left(2\right)},\ldots ,\sigma^{\left(N\right)}$ is the production terms of these fluxes. Then the proper fundamental equation will be the generalization of the classical theory Equation (2)

(27) $$\partial_{t}s=T^{-1}\partial_{t}u-\alpha_{1}q^{\left(1\right)}⊗\partial_{t}q^{\left(1\right)}-\ldots -\alpha_{N}q^{\left(N\right)}⊗\partial_{t}q^{\left(N\right)}$$

where a sign ⊗ means the tensor contraction. By analogy with classical irreversible thermodynamics, the rate of entropy density change is assumed to be representable in the form of a standard equation. In that case, expressing a flux as:

(28) $$J_{s}=T^{-1}q^{\left(1\right)}-\beta_{1}q^{\left(2\right)}\cdot q^{\left(1\right)}-\ldots -\beta_{N}q^{\left(N\right)}⊗q^{\left(N-1\right)}$$

it is easy to obtain the expression for the entropy production:

(29) $$\sigma_{s}=q^{\left(1\right)}\cdot \left[-\alpha_{1}\partial_{t}q^{\left(1\right)}+\beta_{1}\nabla \cdot q^{\left(2\right)}+\nabla T^{-1}\right]+\overset{N}{\underset{m=2}{\sum }}q^{\left(m\right)}⊗[-\alpha_{m}\partial_{t}q^{\left(m\right)}\;+\beta_{m}\nabla \cdot q^{\left(m+1\right)}+\beta_{m-1}\nabla q^{\left(m-1\right)}]$$

where $\sigma_{s}\geq 0$ obeys the second law of thermodynamics. The expressions in square brackets in Equation (29) are the thermodynamic forces, and the simplest phenomenological equations that satisfy non-negativity of the entropy production are the following linear equations:

(30) $$\begin{array}{c} -\alpha_{1}\partial_{t}q^{\left(1\right)}+\beta_{1}\nabla \cdot q^{\left(2\right)}+\nabla T^{-1}=\mu_{1}q^{\left(1\right)}\\ -\alpha_{2}\partial_{t}q^{\left(2\right)}+\beta_{1}\nabla \cdot q^{\left(3\right)}+\beta_{1}\nabla q^{\left(1\right)}=\mu_{2}q^{\left(2\right)}\\ ................................................................\\ -\alpha_{N}\partial_{t}q^{\left(N\right)}+\beta_{N}\nabla \cdot q^{\left(N+1\right)}+\beta_{N-1}\nabla q^{\left(N-1\right)}=\mu_{N}q^{\left(N\right)} \end{array}$$

The coefficients in the phenomenological equations are related to the relaxations times ($\tau_{1}=\alpha_{1}/\mu_{1},\ldots ,\tau_{N}=\alpha_{N}/\mu_{N}$) and correlation lengths ($l_{1}^{2}=\beta_{1}^{2}/\left(\mu_{1}\mu_{2}\right),\ldots ,l_{N}^{2}=\beta_{N}^{2}/\left(\mu_{N}\mu_{N+1}\right)$). In addition to this fact, $\alpha_{1}=-\beta_{1},\ldots ,\alpha_{N}=-\beta_{N}$. Unlike the unique classical theory of Equation (6), the EIT results in the infinite hierarchy of phenomenological equations at N→∞. 

Applying the Fourier’s transform to the phenomenological Equation (26), we have the following expression for the heat flux $\hat{q}\left(\omega ,k\right)$:

(31) $$\hat{q}\left(\omega ,k\right)=-ik\lambda \left(\omega ,k\right)\hat{T}\left(\omega ,k\right)$$

where $\hat{T}\left(\omega ,k\right)$ is the Fourier’s transform of the local-equilibrium temperature, λ(ω,k) is the generalized thermal conductivity coefficient, which can be presented as the continued fraction expansion:

(32) $$\lambda \left(\omega ,k\right)=\frac{\lambda_{0}\left(T\right)}{1+i\omega \tau_{1}+\frac{k^{2}l_{1}^{2}}{1+i\omega \tau_{2}+\frac{k^{2}l_{2}^{2}}{1+i\omega \tau_{3}+\ldots }}}$$

where $\lambda_{0}\left(T\right)$ is the bulk thermal conductivity, k is the wave vector (we are considering a one-dimensional case). It is assumed that all relaxation times and correlation lengths are equal ($\tau_{1}=\ldots =\tau_{N}=\tau_{q}$, $l_{1}=\ldots =l_{N}=l_{q}$) and Equation (32) depends only on one relaxation time $\tau_{q}$ and one correlation length $l_{q}$. Thus, the asymptotic limit is, as follows:

(33) $$\lambda \left(\omega ,k\right)=\lambda_{0}\left(T\right)\frac{-\left(1+i\omega \tau_{q}\right)+\sqrt{{\left(1+i\omega \tau_{q}\right)}^{2}+k^{2}l_{q}^{2}}}{\left(1/2\right)k^{2}l_{q}^{2}}$$

If considering only the steady states (ω→0) and determining the vector k as k=2πL, where L is the characteristic length, then Equation (33) is reduced to the form:

(34) $$\lambda \left(L/l_{q}\right)=\lambda_{0}\left(T\right)\frac{L^{2}}{2\pi^{2}l_{q}^{2}}\left(\sqrt{1+4\pi^{2}\frac{l_{q}^{2}}{L^{2}}}-1\right)$$

Equation (34) is the required dependence of the generalized thermal conductivity on the parameter $\frac{l_{q}}{L}=\mathrm{Kn}$, which is the well-known Knudsen’s number. 

For a stationary state, truncations of Equation (33) are of interest. For example, the second truncation is:

(35) $$\lambda \left(L/l_{q}\right)=\lambda_{0}\left(T\right)f\left(L/l_{q}\right)=\lambda_{0}\left(T\right)\frac{1}{1+\frac{\frac{l_{q}^{2}}{4\pi^{2}L^{2}}}{1+\frac{l_{q}^{2}}{4\pi^{2}L^{2}}}}$$

where $f\left(L/l_{q}\right)$ is the dimensionless function which takes values from zero to one. Thus, in the context of the considered theory with increasing $L/l_{q}$, we have $\lambda \left(L/l_{q}\right)\to \lambda_{0}$, and at $L/l_{q}\to 0$, we have the linear dependence $\lambda \left(L/l_{q}\right)=\lambda_{0}L/\left(\pi l_{q}\right)$. The obtained dependence from Equation (34) describes the experimental data well [16].

## 4. Higher-Order Time Derivatives of Usual Variables

In this Section we will consider a thermodynamic theory stating that the entropy density is a function of both usual thermodynamic variables and their higher-order time derivatives. It can be considered as the postulate of continuous thermodynamics, as well as a consequence of the local uniformity hypothesis [26], which is less strict if comparing with the local-equilibrium hypothesis. According to the local uniformity hypothesis [26], a system that is non-equilibrium as a whole is considered as uniform in each point, and that is why we may increase the number of state variables. For diffusion and chemical reactions without convection, instead of Equation (3) we have [21,22]:

(36) $$s=s\;\left(\rho_{1},\rho_{2},\ldots ,\rho_{K};\partial_{t}\rho_{1},\partial_{t}\rho_{2},\ldots ,\partial_{t}\rho_{K};\ldots \ldots ;\partial_{t^{N-1}}\rho_{1},\partial_{t^{N-1}}\rho_{2},\ldots ,\partial_{t^{N-1}}\rho_{K}\right)$$

where $\partial_{t}\rho_{i}$ is the first-order derivative, $\partial_{t^{2}}\rho_{i}$ is the second-order derivative, …, $\partial_{t^{N-1}}\rho_{i}$ is the (N−1)-th-order derivative, and i=1,2,…,K. 

Based on Equation (36), we write the fundamental equation which is the generalization of Equation (2), as follows:

(37) $$\begin{array}{c} \partial_{t}s=-\underset{i}{\sum }\theta^{-1}{\bar{\mu }}_{i}\partial_{t}\rho_{i}-\underset{i}{\sum }\theta^{-1}\Gamma_{i2}\partial_{t}\partial_{t}\rho_{i}\\ -\underset{i}{\sum }\theta^{-1}\Gamma_{i3}\partial_{t}\partial_{t^{2}}\rho_{i}-\ldots -\underset{i}{\sum }\theta^{-1}\Gamma_{iN}\partial_{t}\partial_{t^{N-1}}\rho_{i} \end{array}$$

where θ is the generalized temperature, ${\bar{\mu }}_{i}$ are the generalized chemical potential i=1,2,…,K, $\Gamma_{i1},\Gamma_{i2},\ldots ,\Gamma_{iN}$ are the new intensive quantities which correspond to the extra variables ($\Gamma_{i1}={\bar{\mu }}_{i}$). All intensive quantities ($\theta ,{\bar{\mu }}_{i},\Gamma_{i2},\Gamma_{i3},\ldots ,\Gamma_{iN},\;i=1,2,\ldots ,K$) depend on both usual and extra variables. The transition to the generalized fundamental Equation (37) means that the local-equilibrium hypothesis is invalid and the thermodynamic system becomes a non-equilibrium as a whole, and at each small element of volume (locally non-equilibrium system). There is meanwhile the thermodynamic formalism identical to classical with preserved basic statements of non-equilibrium thermodynamics [27]. 

In addition to Equation (11), we will use the equations obtained by differentiating the left and right sides of Equation (11) with respect to time:

(38) $$\begin{array}{c} \partial_{t}\partial_{t}\rho_{i}=-\nabla \cdot \left(\partial_{t}J_{i}\right)+\underset{r}{\sum }\nu_{ir}\partial_{t}w_{r},\\ \partial_{t}\partial_{t^{2}}\rho_{i}=-\nabla \cdot \left(\partial_{t^{2}}J_{i}\right)+\underset{r}{\sum }\nu_{ir}\partial_{t^{2}}w_{r},\\ .........................................\\ \partial_{t}\partial_{t^{N-1}}\rho_{i}=-\nabla \cdot \left(\partial_{t^{N-1}}J_{i}\right)+\underset{r}{\sum }\nu_{ir}\partial_{t^{N-1}}w_{r}, \end{array}\;i=1,2,\ldots ,K,\;r=1,2,\ldots ,Q$$

Like classical thermodynamics, the generalized fundamental Equation (37) and the set of balance Equation (38) allow one to construct the formalism of extended irreversible thermodynamics, namely, to obtain the entropy balance equation, to introduce the generalized fluxes and thermodynamic forces, and to establish a relationship between them. In the extended case, the flux and the entropy production instead Equations (15) and (16) are, as follows:

(39) $$J_{s}=\theta^{-1}\underset{i}{\sum }\left({\bar{\mu }}_{i}J_{i}+\Gamma_{i2}{\dot{J}}_{i}+\ldots +\Gamma_{iN}\overset{N-1}{J_{i}}\right)\;\sigma_{s}^{\mathrm{diff}}=\underset{i}{\sum }\left[J_{i}\cdot \nabla \left(\theta^{-1}{\bar{\mu }}_{i}\right)+\left(\partial_{t}J_{i}\right)\cdot \nabla \left(\theta^{-1}\Gamma_{i2}\right)+\ldots +\left(\partial_{t^{N-1}}J_{i}\right)\cdot \nabla \left(\theta^{-1}\Gamma_{iN}\right)\right]\geq 0,\;\sigma_{s}^{\mathrm{react}}=\theta^{-1}\underset{r}{\sum }\left(w_{r}{\bar{A}}_{r}+{\dot{w}}_{r}B_{r2}+\ldots +\overset{N-1}{w_{r}}B_{rN}\right)\geq 0,$$

where ${\bar{A}}_{r}=-\sum_{i}\nu_{ir}{\bar{\mu }}_{i}$ is the generalized affinity, $B_{rj}=-\sum_{i}\nu_{ir}\Gamma_{ij}$ is the analog of chemical affinity corresponding to variables $\partial_{t^{j-1}}\rho_{1},\partial_{t^{j-1}}\rho_{2},\ldots ,\partial_{t^{j-1}}\rho_{K}$ (j=1,2,…,N; at j=1 we have $B_{1r}={\bar{A}}_{r}$). As the result, we obtain a set of phenomenological equations which connect thermodynamic forces $\nabla \left(\theta^{-1}{\bar{\mu }}_{i}\right)$, $\nabla \left(\theta^{-1}\Gamma_{i2}\right)$, $\nabla \left(\theta^{-1}\Gamma_{i3}\right)$, …, $\nabla \left(\theta^{-1}\Gamma_{iN}\right)$ and fluxes $J_{i},\partial_{t}J_{i},\partial_{t^{2}}J_{i},\ldots ,\partial_{t^{N-1}}J$, as well as $\theta^{-1}{\bar{A}}_{r},\;\theta^{-1}B_{2r}+\ldots +\theta^{-1}B_{Nr}$ and $w_{r},\;\partial_{t}w_{r},\ldots ,\partial_{t^{N-1}}w_{r}$. In the simplest case these quantities are connected by the linear phenomenological equations, which ensure the positivity of entropy production. For the diffusion process, we have:

(40) $$\begin{array}{c} \nabla \left(\theta^{-1}{\bar{\mu }}_{i}\right)=R_{11}^{\left(i\right)}\;J_{i}+R_{12}^{\left(i\right)}\;\partial_{t}J_{i}+\ldots +R_{1N}^{\left(i\right)}\;\partial_{t^{N-1}}J_{i}\\ \nabla \left(\theta^{-1}\Gamma_{i2}\right)=R_{21}^{\left(i\right)}\;J_{i}+R_{22}^{\left(i\right)}\;\partial_{t}J_{i}+\ldots +R_{2N}^{\left(i\right)}\partial_{t^{N-1}}J_{i}\\ .......................................................\\ \nabla \left(\theta^{-1}\Gamma_{iN}\right)=R_{N1}^{\left(i\right)}\;J_{i}+R_{N2}^{\left(i\right)}\;\partial_{t}J_{i}+\ldots +R_{NN}^{\left(i\right)}\;\partial_{t^{N-1}}J_{i} \end{array},\;i=1,\;2,\;\ldots ,\;K,\ldots$$

where $R_{\alpha \beta }^{\left(i\right)}$ are the phenomenological coefficients, α,β=1,2,…,N. The similar equations can be obtained for the chemical reactions:

(41) $$\begin{array}{c} \theta^{-1}{\bar{A}}_{r}=\lambda_{11}^{\left(r\right)}w_{r}+\lambda_{12}^{\left(r\right)}\partial_{t}w_{r}+\lambda_{13}^{\left(r\right)}\partial_{t^{2}}w_{r}w_{r}+\ldots +\lambda_{1N}^{\left(r\right)}\partial_{t^{N-1}}w_{r}\\ \theta^{-1}B_{r2}=\lambda_{21}^{\left(r\right)}w_{r}+\lambda_{22}^{\left(r\right)}\partial_{t}w_{r}+\lambda_{23}^{\left(r\right)}\partial_{t^{2}}w_{r}+\ldots +\lambda_{2N}^{\left(r\right)}\partial_{t^{N-1}}w_{r}\\ ....................................................\\ \theta^{-1}B_{rN}=\lambda_{N1}^{\left(r\right)}w_{r}+\lambda_{N2}^{\left(r\right)}\partial_{t}w_{r}+\lambda_{N3}^{\left(r\right)}\partial_{t^{2}}w_{r}+\ldots +\lambda_{NN}^{\left(r\right)}\partial_{t^{N-1}}w_{r} \end{array}\;r=1,\;2,\;\ldots ,\;Q,$$

where $\lambda_{\delta \gamma }^{\left(r\right)}$ are the phenomenological coefficients, δ,γ=1,2,…,N. Thus, for the extended case K Equation (17) are substituted with NK phenomenological Equation (40), and Q Equation (18) are replaced with NQ Equation (41). 

## 5. Two-Dimensional Case of Phenomenological Equations

Let us further focus on the case N=2. Then the set of Equation (40) can be written in a much simpler form:

(42) $$\nabla \left(\theta^{-1}{\bar{\mu }}_{i}\right)=R_{11}^{\left(i\right)}\;J_{i}+R_{12}^{\left(i\right)}\;\partial_{t}J_{i}$$

(43) $$\nabla \left(\theta^{-1}\Gamma_{i2}\right)=R_{21}^{\left(i\right)}\;J_{i}+R_{22}^{\left(i\right)}\;\partial_{t}J_{i}.$$

It is well known that in EIT the Onsager-Casimir reciprocal relations [4,5] have not been proven. However, we believe that there is the following basis for proving reciprocal relations out of local equilibrium:

(i) the proposed theory is based on the local homogeneity hypothesis, which is a less rigorous analogue of the local equilibrium hypothesis;
(ii) the classical Onsager-Machlup theory of fluctuations is applicable for systems with memory;
(iii) the additional variables considered in the proposed theory are independent variables.

We further assume that Onsager-Casimir reciprocal relations take place in the extended theory. It is obvious that fluxes $J_{i}$ are odd variables. Hence time derivatives $\partial_{t}J_{i}$ should be viewed as even variables. Therefore, the matrix of phenomenological coefficients in Equations (42) and (43) are untisimmetric, i.e., $R_{21}^{\left(i\right)}=-R_{12}^{\left(i\right)}$.

Let us divide Equation (42) by a factor $R_{11}^{\left(i\right)}$ and make the following substitution by analogy with classical thermodynamics (Equation (19)):

$$\tau_{i}\;\partial_{t}J_{i}+J_{i}=\frac{1}{R_{11}^{\left(i\right)}}\nabla \left(\theta^{-1}{\bar{\mu }}_{i}\right)=-D_{0i}\nabla n_{i}$$

(here, the generalized temperature and chemical potentials are assumed to depend on the component concentrations). Moreover, we imply that $R_{12}^{\left(i\right)}/R_{11}^{\left(i\right)}=\tau_{i}$, where $\tau_{i}$ is the relaxation time. Therefore, Equation (42) can be written as:

(44) $$\tau_{i}\;\partial_{t}J_{i}+J_{i}=-D_{0i}\nabla n_{i}$$

being the Maxwell-Cattaneo Equation [11]. We further assume that $\theta^{-1}\Gamma_{i2}$ are the homogeneous quantities; i.e., $\nabla \left(\theta^{-1}\Gamma_{i2}\right)=0$, and the diffusion of the components is defined by only the gradient $\nabla \left(\theta^{-1}{\bar{\mu }}_{i}\right)$. Divide the second equation by the factor $R_{11}^{\left(i\right)}$ and introduce the characteristic time of change in macroscopic parameters $\tau_{0}{}^{2}=R_{22}^{\left(i\right)}/R_{11}^{\left(i\right)}$, where $\tau_{0}$ shows no dependence on a certain component *i*. Then the system of Equations (42) and (43) can also be simplified as:

(45) $$-D_{0i}\nabla n_{i}=J_{i}+\tau_{i}\partial_{t}J_{i}$$

(46) $$0=-\tau_{i}J_{i}+\tau_{0}{}^{2}\partial_{t}J_{i}$$

that allows it to be written in the matrix form:

(47) $$-L_{2}^{0}D_{0i}\nabla n_{i}=M_{2}\cdot J_{2},$$

where:

(48) $$L_{2}^{0}=\left(\begin{array}{c} 1\\ 0 \end{array}\right),\;M_{2}=\left(\begin{array}{cc} 1 & \tau_{i}\\ -\tau_{i} & \tau_{0}^{2} \end{array}\right),\;J_{2}=\left(\begin{array}{c} J_{i}\\ \partial_{t}J_{i} \end{array}\right).$$

As is seen, Equation (44) is the generalized Fick’s law (19) with adding a term $\tau_{i}\;\partial_{t}J_{i}$. In this case, the gradient $\nabla n_{i}$ induces both the diffusion flux $J_{i}$ and the rate of its change $\partial_{t}J_{i}$. Equation (44) leads to a delay in the propagation of diffusing components compared to classical thermodynamics. The greater is the relaxation time $\tau_{i}$, the further is the thermodynamic system from the local-equilibrium state. 

By solving a system of Equations (45) and (46) relative to $J_{i}$ and $\partial_{t}J_{i}$ using the Kramers formula, we get the following expressions for the flux and its time derivative:

(49) $$J_{i}=-\frac{\left|{\tilde{M}}_{2}\right|}{\left|M_{2}\right|}D_{0i}\nabla n_{i}=-f_{2}\left(\tau_{i},\tau_{0}\right)D_{0i}\nabla n_{i},\;\tau_{i}\partial_{t}J_{i}=-\frac{\tau_{i}\left|{\tilde{\tilde{M}}}_{2}\right|}{\left|M_{2}\right|}D_{0i}\nabla n_{i}=-g_{2}\left(\tau_{i},\tau_{0}\right)D_{0i}\nabla n_{i}$$

where ${\tilde{M}}_{2}=\left(\begin{array}{cc} 1 & \tau_{i}\\ 0 & \tau_{0}^{2} \end{array}\right)$, ${\tilde{\tilde{M}}}_{2}=\left(\begin{array}{cc} 1 & 1\\ -\tau_{i} & 0 \end{array}\right)$.

(50) $$f_{2}\left(\tau_{i},\tau_{0}\right)=\frac{\left|{\tilde{M}}_{2}\right|}{\left|M_{2}\right|}=\frac{\tau_{0}{}^{2}}{\tau_{i}{}^{2}+\tau_{0}{}^{2}},\;g_{2}\left(\tau_{i},\tau_{0}\right)=\frac{\tau_{i}\left|{\tilde{\tilde{M}}}_{2}\right|}{\left|M_{2}\right|}=\frac{\tau_{i}{}^{2}}{\tau_{i}{}^{2}+\tau_{0}{}^{2}}$$

at that $f_{2}\left(\tau_{i},\tau_{0}\right)+g_{2}\left(\tau_{i},\tau_{0}\right)=1$. Again, by comparing the first equality in Equation (49) with Fick’s law, we get the diffusion coefficient $D_{0i}$ multiplied by the function $f\left(\tau_{i},\tau_{0}\right)$ in the extended theory, which takes values from zero to one. In this connection, one can introduce a generalized diffusion coefficient:

(51) $$D_{i}=D_{0i}f\left(\tau_{i},\tau_{0}\right)=D_{0i}\frac{\tau_{0}{}^{2}}{\tau_{i}{}^{2}+\tau_{0}{}^{2}}$$

The greater is the memory effect in the non-equilibrium system, i.e., the greater is $\tau_{i}$, the lower are the diffusion flux $J_{i}$ in Equation (49) and the generalized diffusion coefficient $D_{i}$ in Equation (51). Therefore, the memory effect reduces the diffusion coefficient and, consequently, the rate of diffusion of components. In some cases diffusion may occur in the form of waves [11]. 

The similar results are obtained for the rate of chemical reaction. In a two-dimensional case (*N* = 2) a set of Equation (41) is also reduced to two equations:

(52) $$\theta^{-1}{\bar{A}}_{r}=\lambda_{11}^{\left(r\right)}w_{r}+\lambda_{12}^{\left(r\right)}\partial_{t}w_{r},$$

(53) $$\theta^{-1}B_{r2}=\lambda_{21}^{\left(r\right)}w_{r}+\lambda_{22}^{\left(r\right)}\partial_{t}w_{r},$$

where $\lambda_{12}^{\left(r\right)}=-\lambda_{21}^{\left(r\right)}$. Let us divide the left and right sides of Equation (52) by the factor $\lambda_{11}^{\left(r\right)}$ and introduce the relaxation time for a chemical reaction: $\tau_{r}=\lambda_{12}^{\left(r\right)}/\lambda_{11}^{\left(r\right)}$. Taking into account Equation (23), i.e., that $\lambda_{11}^{\left(r\right)}=\frac{ℜ}{w_{r}^{+}}$, we get the following equation from Equation (52):

(54) $$\tau_{r}\partial_{t}w_{r}+w_{r}=\frac{w_{r}^{+}}{ℜ}\frac{{\bar{A}}_{r}}{\theta }$$

Equation (54) describes the changes over time in the rate of reaction r taking into account the memory effect. By comparing Equations (22) and (54), it is evident that the linear equality (22) is completed with a term $\tau_{r}\partial_{t}w_{r}$. Then, as for diffusion, we assume $B_{r2}=0$ and divide Equation (53) by a factor $\lambda_{11}^{\left(r\right)}$. It is evident that the ratio $\lambda_{22}^{\left(r\right)}/\lambda_{11}^{\left(r\right)}$ is associated with the same characteristic time of changes in macroscopic parameters $\tau_{0}$, i.e., for a chemical reaction $\tau_{0}{}^{2}=\lambda_{22}^{\left(r\right)}/\lambda_{11}^{\left(r\right)}$. Thus, a set of Equations (52) and (53) can be written as:

(55) $$\frac{w_{r}^{+}}{ℜ}\frac{{\bar{A}}_{r}}{\theta }=w_{r}+\tau_{r}\partial_{t}w_{r}$$

(56) $$0=-\tau_{r}w_{r}+\tau_{0}{}^{2}\partial_{t}w_{r}$$

and represented in a matrix notation:

(57) $$L_{2}^{0}\frac{w_{r}^{+}}{ℜ}\frac{{\bar{A}}_{r}}{\theta }=M_{2}\cdot J_{2},$$

where:

$$L_{2}^{0}=\left(\begin{array}{c} 1\\ 0 \end{array}\right),\;M_{2}=\left(\begin{array}{cc} 1 & \tau_{r}\\ -\tau_{r} & \tau_{0}^{2} \end{array}\right),\;J_{2}=\left(\begin{array}{c} w_{r}\\ \partial_{t}w_{r} \end{array}\right).$$

Solving the linear system of Equations (52) and (53) with respect to $w_{r},\partial_{t}w_{r}$, we get the expressions identical to those for diffusion:

(58) $$w_{r}=f_{2}\left(\tau_{r},\tau_{0}\right)\frac{w_{r}^{+}{\bar{A}}_{r}}{ℜ\theta },\;\tau_{r}\partial_{t}w_{r}=g_{2}\left(\tau_{r},\tau_{0}\right)\frac{w_{r}^{+}{\bar{A}}_{r}}{ℜ\theta },$$

where:

(59) $$f_{2}\left(\tau_{r},\tau_{0}\right)=\frac{\left|{\tilde{M}}_{2}\right|}{\left|M_{2}\right|}=\frac{\tau_{0}{}^{2}}{\tau_{r}{}^{2}+\tau_{0}{}^{2}},\;{\tilde{M}}_{2}=\left(\begin{array}{cc} 1 & \tau_{r}\\ 0 & \tau_{0}^{2} \end{array}\right),$$

$$g_{2}\left(\tau_{r},\tau_{0}\right)=\frac{\tau_{r}\left|{\tilde{\tilde{M}}}_{2}\right|}{\left|M_{2}\right|}=\frac{\tau_{r}^{2}}{\tau_{r}^{2}+\tau_{0}^{2}},\;{\tilde{\tilde{M}}}_{2}=\left(\begin{array}{cc} 1 & 1\\ -\tau_{r} & 0 \end{array}\right).$$

Taking into consideration the expression for the rate of direct reaction $w_{r}^{+}=k_{0r}^{+}n_{1}{}^{\nu_{1r}}n_{2}{}^{\nu_{2r}}\ldots n_{L}{}^{\nu_{Lr}}$, we introduce the generalized rate constant for:

(60) $$k_{r}^{+}=k_{0r}^{+}f\left(\tau_{r},\tau_{0}\right)=k_{0r}^{+}\frac{\tau_{0}{}^{2}}{\tau_{r}{}^{2}+\tau_{0}{}^{2}},$$

where the function $f\left(\tau_{r},\tau_{0}\right)$ takes values from zero to one. A similar expression is valid for the rate constant for the reverse reaction $k_{r}^{-}$, as well. Comparing the first expression in Equation (58) with a classical one (Equation (22)), it is obvious that the greater is the relaxation time $\tau_{r}$ (i.e., the further is the system from the local-equilibrium state), the more is delayed the chemical reaction and the lower are the rates of direct and reverse reactions. Therefore, processes with delay reduce the diffusion coefficients, as well as the rate constant of chemical reactions. 

## 6. Characteristic Quantities of Nanosystems

The rates of diffusion and chemical reaction depend not only on $\tau_{i}$, $\tau_{r}$, and $\tau_{0}$. It is well known [8,9,10] that the relaxation time of each component $\tau_{i}$ is related to the characteristic size of microstructure of the medium $l_{i}$ and to the rate of perturbation propagation in the medium $V_{0i}$ (the rate of diffusion wave propagation): $\tau_{i}=l_{i}/V_{0i}$. The same dependence is valid for a chemical reaction: $\tau_{r}=l_{r}/V_{0r}$, where $l_{r}$ is the characteristic microscale corresponding to the chemical reaction, $V_{0r}$ is the rate of approach to equilibrium due to the chemical reaction. The characteristic time $\tau_{0}$ is related to the intrinsic macroscale of the medium L and to the rate of change in macroparameters on account of external effects V: $\tau_{0}=L/V$ (all these quantities depend neither on the nature of components nor on the chemical reaction). Then:

(61) $$\frac{\tau_{0}}{\tau_{i}}=\frac{L}{l_{i}}\frac{V_{0i}}{V},\;\frac{\tau_{0}}{\tau_{r}}=\frac{L}{l_{r}}\frac{V_{0r}}{V},\;i=1,2,\ldots ,K,\;r=1,2,\ldots ,Q$$

It is evident from Equation (61) that the time ratio $\tau_{0}/\tau_{i}$ linearly depends on two dimensionless magnitudes: the linear parameter ratio $L/l_{i}$ and the rate ratio $V_{0i}/V$. In a similar way, $\tau_{0}/\tau_{r}$ depends on $L/l_{r}$ and $V_{0r}/V$. Once $L>>l_{i}$ at a fixed rate ratio $V_{0i}/V$ or $V<<V_{0i}$ at a fixed linear parameter ratio $L/l_{i}$, the thermodynamic system tends to local equilibrium. Otherwise ($L\leq l_{i}$ at a fixed rate ratio or $V\geq V_{0i}$ at a fixed linear parameter ratio), the latter is non-equilibrium. Similar regularities are observed for parameters of the chemical reaction. 

In this paper, the quantity L is a linear size of nanoparticles. We are interested to know whether there is a relationship between the parameters $L/l_{i}$ and $L/l_{r}$ and the rate of diffusion of components in the nanoparticles and the rate of chemical reactions in nanoparticles. In this case $V_{0i}/V=\mathrm{const}$ and $V_{0r}/V=\mathrm{const}$, and we assume for simplicity that $V_{0i}/V=1$ and $V_{0r}/V=1$ and will consider the dependence of the time ratio $\tau_{0}/\tau_{i}$ and $\tau_{0}/\tau_{r}$ on only the parameters $L/l_{i}$ and $L/l_{r}$, respectively:

(62) $$\frac{\tau_{0}}{\tau_{i}}=\frac{L}{l_{i}},\;\frac{\tau_{0}}{\tau_{r}}=\frac{L}{l_{r}},\;i=1,2,\ldots ,K$$

Using Equation (62), we present the diffusion coefficient (51) and the rate constant of the direct reaction (Equation (60)) in the form:

(63) $$D_{i}=D_{0i}f\left(\frac{L}{l_{i}}\right)=D_{0i}\frac{\frac{L^{2}}{l_{i}{}^{2}}}{1+\frac{L^{2}}{l_{i}{}^{2}}},\;i=1,2,\ldots ,K$$

(64) $$k_{r}^{+}=k_{0r}^{+}f\left(\frac{L}{l_{r}}\right)=k_{0r}^{+}\frac{\frac{L^{2}}{l_{r}{}^{2}}}{1+\frac{L^{2}}{l_{r}{}^{2}}},\;r=1,2,\ldots ,Q$$

where $f\left(L/l_{i}\right)=\frac{\frac{L^{2}}{l_{i}{}^{2}}}{1+\frac{L^{2}}{l_{i}{}^{2}}}$ and $f\left(L/l_{r}\right)=\frac{\frac{L^{2}}{l_{r}{}^{2}}}{1+\frac{L^{2}}{l_{r}{}^{2}}}$. Equations (63) and (64) are the first result of the proposed theory. These are the simplest functions obtained by solving a system of linear phenomenological equations. The value L, which is the particle size, and the characteristic sizes of microstructures, $l_{i}$ and $l_{r}$, determine the rates of the processes. As is seen from the Equations (63) and (64) the finer are the nanoparticles (i.e., the lower is L at the constant medium parameters $l_{i}$ and $l_{r}$), the lower are the values of functions $f\left(L/l_{i}\right)$ and $f\left(L/l_{r}\right)$; i.e., the rates of diffusion of components and chemical reactions decrease. Thus, the highest diffusion coefficient and the rate of chemical reaction are observed in sufficiently large particles.

Equation (61) allow the rates of irreversible processes to be established not only as a function of size of a nanosystem. If considering solidification of the undercooled melt, then the length ratio $L/l_{i}$ must be fixed and the diffusion coefficients have to be studied as a function of the rate ratio $V/V_{0i}$, where V is the velocity of propagation of the solidification front of the undercooled melt [9,29,30,31].

## 7. Multi-Dimensional Case

First of all, consider the general case of phenomenological equations at N>2 in assuming that diffusion and chemical reactions are still described by the Maxwell-Cattaneo Equations (44) and (54). Then the matrix equations, which are a generalization of Equations (47) and (57), are being written as:

(65) $$-L_{N}^{0}D_{0i}\nabla n_{i}=M_{N}\cdot J_{N},\;\mathrm{and}\;L_{N}^{0}\frac{w_{r}^{+}}{ℜ}\frac{{\bar{A}}_{r}}{\theta }=M_{N}\cdot J_{N},$$

where the vector $L_{N}^{0}$ consists of the components (1, 0, …, 0), the vector $J_{N}$ includes the components $\left(J_{i},\partial_{t}J_{i},\partial_{t^{2}}J_{i},\ldots ,\partial_{t^{N-1}}J_{i}\right)$ or $\left(w_{r},\partial_{t}w_{r},\partial_{t^{2}}w_{r},\ldots ,\partial_{t^{N-1}}w_{r}\right)$, and the matrix $M_{N}$ depends on the relaxation times $\tau_{0}$ and $\tau_{}$, where $\tau_{}$ denotes $\tau_{i}$ or $\tau_{r}$. We have therefore omitted the indices i and r in the matrix $M_{N}$. 

If basing on the Maxwell-Cattaneo constitutive equation, then all coefficients starting from the third in the first row of the matrix $M_{N}$ composed of phenomenological coefficients of Equations (40) and (41) vanish. Thus, the first row of the matrix $M_{N}$ has a form (1,τ,0,…,0), and the first column in $M_{N}$ is written as (1,−τ,0,…,0). We further assume that the diagonal coefficients in $M_{N}$ depend on only $\tau_{0}$, while the non-diagonal ones are the functions of τ. That’s why a three-dimensional case is presented by the following expressions:

(66) $$M_{3}=\left(\begin{array}{ccc} 1 & \tau & 0\\ -\tau & \tau_{0}^{2} & \tau \\ 0 & -\tau & 1 \end{array}\right)\;\mathrm{and}\;f_{3}\left(\tau ,\tau_{0}\right)=\frac{\left|{\tilde{M}}_{3}\right|}{\left|M_{3}\right|}=\frac{1}{1+\frac{a^{2}}{1+a^{2}}},\;\mathrm{where}\;a=\frac{\tau }{\tau_{0}}.$$

As is seen from Equation (66), we again assume that the Onsager-Casimir reciprocity relations are valid. A function $f_{3}\left(\tau ,\tau_{0}\right)$ is the second-order truncation of continuous-fraction expansion, which can be presented in the form:

(67) $$f_{N\to \infty }\left(\tau ,\tau_{0}\right)=\frac{1}{1+\frac{a^{2}}{1+\frac{a^{2}}{1+\frac{a^{2}}{1+\ldots }}}},\;a=\frac{\tau }{\tau_{0}}.$$

Similarly for all odd N, if a function $f_{N}\left(\tau ,\tau_{0}\right)$ is representable as finite truncation of Equation (67), it must be assumed that the matrix $M_{N}$ satisfies the Onsager-Casimir relations. As in traditional version of EIT, at $\tau_{0}/\tau \to 0$ the function $f_{N}\left(\tau ,\tau_{0}\right)$ tends to a non-vanishing limit. At N→∞ for $f_{N}\left(\tau ,\tau_{0}\right)$ we have the asymptotic function:

(68) $$f_{N\to \infty }\left(\tau ,\tau_{0}\right)=\frac{\tau_{0}^{2}}{2\tau^{2}}\left(\sqrt{1+\frac{4\tau^{2}}{\tau_{0}^{2}}}-1\right).$$

**Figure 1:**
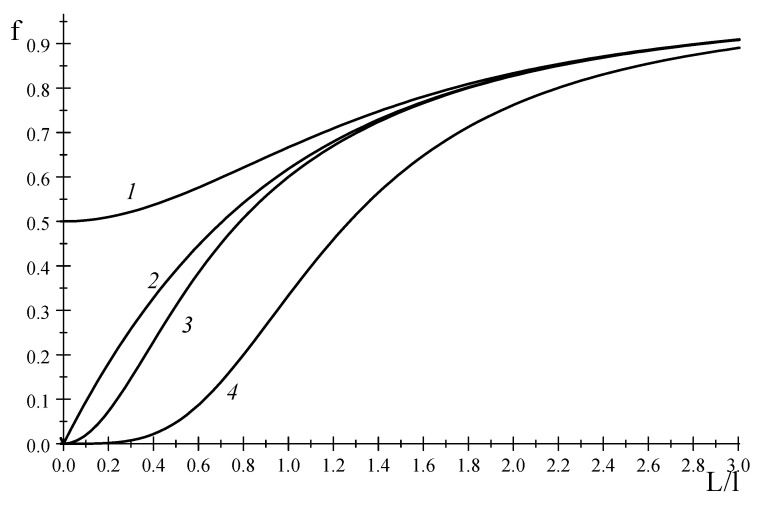
Behavior of dimensionless function f in terms of ratio L/l: 1—Equation (66), 2—Equations (67) or (68), 3—Equation (69), and 4—Equation (70).

In the present work we are interested in a relationship between the nanoparticle dispersity and the rate of irreversible processes which occur in nanoparticles. In this connection, we focus on the case when $\tau_{0}/\tau =L/l$ (Equation (62)). Figure 1 displays a typical function at odd N=3 and an asymptotical function $f_{N\to \infty }$ depending on L/l.

Then at even N consider $M_{N}$ which results in a function $f_{N}\left(\tau ,\tau_{0}\right)$ as the truncation of continuous fraction expansion. Nevertheless, for this condition at N>3 we have the matrices, for which the Onsager-Casimir reciprocal relations are invalid. For instance, at N=4:

(69) $$M_{4}=\left(\begin{array}{cccc} 1 & \tau & 0 & 0\\ -\tau & \tau_{0}^{2} & 0 & \tau \\ 0 & 0 & \tau_{0}^{2} & \tau \\ 0 & -\tau & -\tau & 1 \end{array}\right),\;f_{4}\left(\tau ,\tau_{0}\right)=\frac{1}{1+\frac{a^{2}}{1+\frac{a^{2}}{1+a^{2}}}},$$

where the function $f_{4}\left(\tau ,\tau_{0}\right)$ is representable as truncation of continued-fraction expansion. The matrix $M_{N}$ is easily transformed into the matrix $M_{N}^{*}$ (by replacing a sign before some τ), for which the Onsager-Casimir reciprocal relations hold. However, in this case the function $M_{N}$ cannot be presented as truncation of continued-fraction expansion. For example, at N=4 the replacement of the sign before τ in the fourth row of the matrix $M_{4}$ gives:

(70) $$M_{4}^{*}=\left(\begin{array}{cccc} 1 & \tau & 0 & 0\\ -\tau & \tau_{0}^{2} & 0 & \tau \\ 0 & 0 & \tau_{0}^{2} & \tau \\ 0 & \tau & -\tau & 1 \end{array}\right),\;f_{4}^{*}\left(\tau ,\tau_{0}\right)=\frac{1}{1+a^{2}+a^{4}},$$

Figure 1 displays the functions $f_{4}$ and $f_{4}^{*}$. As is seen in Figure 1, $f_{4}$ is closer to the asymptotic limit, rather than $f_{4}^{*}$. A similar regularity is observed for all even N.

Consider now the general case N>2, when the constitutive equation may contain up to N−1 time derivatives of fluxes and depends on one relaxation time τ:

(71) $$\tau^{N-1}\partial_{t^{N-1}}J_{i}+,\ldots ,+\tau^{2}\partial_{t^{2}}J_{i}+\tau \partial_{t}J_{i}+J_{i}=-D_{0}\nabla n_{i}$$

or:

(72) $$\tau^{N-1}\partial_{t^{N-1}}w_{r}+,\ldots ,+\tau^{2}\partial_{t^{2}}w_{r}+\tau \partial_{t}w_{r},+w_{r}=\frac{w_{r}^{+}}{ℜ}\frac{\bar{A}}{\theta }.$$

Let N=3 and constitutive Equations (71) and (72) contain the time derivatives of flux up to the second order inclusive. For this, we replace the first row in the matrix $M_{3}$ (Equation (66)) with $\left(1,\tau ,\tau^{2}\right)$ and the first column with $\left(1,-\tau ,+\tau^{2}\right)$. In addition to this, let the degree τ or $\tau_{0}$ in each subsequent element of the row or the column in the matrix $M_{3}$ be higher that the degree τ or $\tau_{0}$ of the previous element by one. Then the obtained matrix $M_{3}^{\left(2\right)}$ and the function $f_{3}^{\left(2\right)}\left(\tau ,\tau_{0}\right)$ will take the form:

(73) $$M_{3}^{\left(2\right)}=\left(\begin{array}{ccc} 1 & \tau & \tau^{2}\\ -\tau & \tau_{0}^{2} & \tau^{3}\\ \tau^{2} & -\tau^{3} & \tau_{0}^{4} \end{array}\right),\;f_{3}^{\left(2\right)}\left(\tau ,\tau_{0}\right)=\frac{1+a^{6}}{1+a^{2}-a^{4}+3a^{6}},\;a=\frac{\tau }{\tau_{0}}.$$

**Figure 2:**
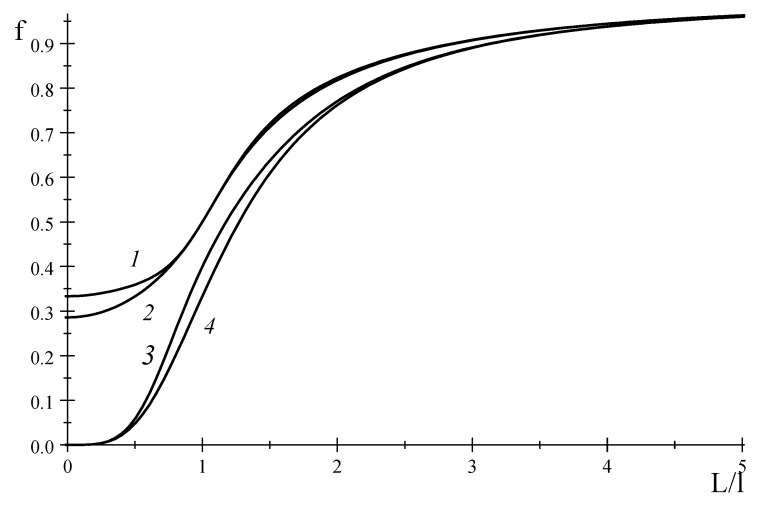
Behavior of dimensionless function f in terms of ratio L/l: 1—Equation (73), 2—Equation (74), 3—Equation (75), and 4—Equation (76).

Similarly, the matrix $M_{4}^{\left(3\right)}$ and the function $f_{4}^{\left(3\right)}\left(\tau ,\tau_{0}\right)$ can be obtained instead of Equation (73):

(74) $$M_{4}^{\left(3\right)}=\left(\begin{array}{cccc} 1 & \tau & \tau^{2} & \tau^{3}\\ -\tau & \tau_{0}^{2} & \tau^{3} & \tau^{4}\\ \tau^{2} & -\tau^{3} & \tau_{0}^{4} & \tau^{5}\\ -\tau^{3} & \tau^{4} & -\tau^{5} & \tau_{0}^{5} \end{array}\right),\;f_{4}^{\left(3\right)}\left(\tau ,\tau_{0}\right)=\frac{1-a^{2}+a^{4}-a^{8}+2a^{10}}{1-a^{4}+5a^{6}-8a^{8}+7a^{10}},$$

where we assumed that $M_{3}^{\left(2\right)}$ and $M_{4}^{\left(3\right)}$ satisfy the Onsager-Casimir relations. Figure 2 shows the functions $f_{3}^{\left(2\right)}\left(L/l\right)$ and $f_{4}^{\left(3\right)}\left(L/l\right)$ that seem to tend to a finite value at L/l→0. Meanwhile, if some coefficients in these matrices are assumed to be zero, we obtain the functions $f_{3}^{\left(2\right)}\left(L/l\right)$ and $f_{4}^{\left(3\right)}\left(L/l\right)$ which tend to zero at L/l→0. This is evident from Figure 2 at:

(75) $$M_{4}^{\left(3\right)}=\left(\begin{array}{cccc} 1 & \tau & \tau^{2} & \tau^{3}\\ -\tau & \tau_{0}^{2} & 0 & \tau^{4}\\ \tau^{2} & 0 & \tau_{0}^{4} & 0\\ -\tau^{3} & \tau^{4} & 0 & \tau_{0}^{5} \end{array}\right),\;f_{4}^{\left(3\right)}\left(\tau ,\tau_{0}\right)=\frac{1+a^{4}}{1+a^{2}+2a^{4}+a^{8}}$$

(76) $$M_{3}^{\left(2\right)}=\left(\begin{array}{ccc} 1 & \tau & \tau^{2}\\ -\tau & \tau_{0}^{2} & 0\\ \tau^{2} & 0 & \tau_{0}^{4} \end{array}\right),\;f_{3}^{\left(2\right)}\left(\tau ,\tau_{0}\right)=\frac{1}{1+a^{2}+a^{4}}.$$

We can see that Equations (73)–(76) cannot be representable as truncations of continued-fraction expansion.

## 8. Constitutive Equations

In this section, we consider some constitutive equations that describe the diffusion process in nanosystems. It should be noted that even in a two-dimensional case the obtained constitutive equations are not limited by the Maxwell-Cattaneo Equation (44) and equations containing the higher-order time derivatives of diffusion fluxes in Equations (71) and (72). Let us consider a case when the generalized temperature θ and chemical potential ${\bar{\mu }}_{i}$ depend on $n_{i}$ and $\partial_{t}n_{i}$. Then the thermodynamic force $\nabla \left(\theta^{-1}{\bar{\mu }}_{i}\right)$ is written as:

(77) $$\nabla \left(\theta^{-1}{\bar{\mu }}_{i}\right)=\frac{\partial \left(\theta^{-1}{\bar{\mu }}_{i}\right)}{\partial n_{i}}\nabla n_{i}+\frac{\partial \left(\theta^{-1}{\bar{\mu }}_{i}\right)}{\partial \partial_{t}n_{i}}\nabla \partial_{t}n_{i}.$$

Using Equation (77), we make a replacement in equation:

(78) $$\tau_{i}\;\partial_{t}J_{i}+J_{i}=\frac{1}{R_{11}^{\left(i\right)}}\nabla \left(\theta^{-1}{\bar{\mu }}_{i}\right)$$

that results in the Jeffreys type constitutive equation (dual-phase-lag transfer equation [22,25]) instead of Equation (44), which describes the diffusion of components:

(79) $$\tau_{i}\;\partial_{t}J_{i}+J_{i}=-D_{0i}\nabla \left(n_{i}+\eta \partial_{t}n_{i}\right),$$

where $D_{0i}=\frac{1}{R_{11}^{\left(i\right)}}\frac{\partial \left(\theta^{-1}{\bar{\mu }}_{i}\right)}{\partial n_{i}}$, $\eta =\frac{\partial \left(\theta^{-1}{\bar{\mu }}_{i}\right)}{\partial \partial_{t}n_{i}}{\left[\frac{\partial \left(\theta^{-1}{\bar{\mu }}_{i}\right)}{\partial n_{i}}\right]}^{-1}$. 

Further, using the integral representation of the diffusion flux, we obtain the higher-order diffusion equations. Let us consider the expression [32,33] for the diffusion flux:

(80) $$J_{i}\left(t\right)=-\kappa \int_{0}^{\infty }\exp\left(-\frac{t^{\prime }}{\tau_{i}}\right)\nabla n_{i}\left(t-t^{\prime }\right)dt^{\prime },$$

where κ is a constant. Let us represent the right-hand side of the Expression (80) in the form

(81) $$J_{i}\left(t\right)=-\tau \kappa \nabla n_{i}\left(t\right)+\tau \kappa \int_{0}^{\infty }\exp\left(-\frac{t^{\prime }}{\tau_{i}}\right)\partial_{t}\nabla n_{i}\left(t-t^{\prime }\right)dt^{\prime }.$$

Using (80) we obtain from (81) the Maxwell-Cattaneo type equation:

(82) $$J_{i}\left(t\right)=-\tau \kappa \nabla n_{i}\left(t\right)-\tau \partial_{t}J_{i}\left(t\right),$$

where $\tau \kappa =D_{0i}$ Similarly, from (81) we can obtain the equality:

(83) $$J_{i}\left(t\right)=-\tau \kappa \nabla n_{i}\left(t\right)+\tau^{2}\kappa \partial_{t}\nabla n_{i}\left(t\right)-\tau^{3}\kappa \partial_{t^{2}}\nabla n_{i}\left(t\right)+\;\tau^{3}\kappa \int_{0}^{\infty }\exp\left(-\frac{t^{\prime }}{\tau_{i}}\right)\partial_{t^{3}}\nabla n_{i}\left(t-t^{\prime }\right)dt^{\prime },$$

which can be cast into equation:

(84) $$\tau_{i}^{3}\;\partial_{t^{3}}J_{i}+J_{i}=-D_{0i}\nabla \left(n_{i}-\tau_{i}\partial_{t}n_{i}+\tau_{i}^{2}\partial_{t^{2}}n_{i}\right).$$

The comparison of Equations (84) with (78) allows us to write the right-hand side of Equation (84) in the form $\left(1/R_{11}^{\left(i\right)}\right)\nabla \left(\theta^{-1}{\bar{\mu }}_{i}\right)$. As a result, we obtain the corresponding phenomenological equation:

(85) $$\nabla \left(\theta^{-1}{\bar{\mu }}_{i}\right)=R_{11}^{\left(i\right)}J_{i}+R_{11}^{\left(i\right)}\tau_{i}^{3}\;\partial_{t^{3}}J_{i},$$

where:

(86) $$\theta^{-1}{\bar{\mu }}_{i}=n_{i}-\tau_{i}\partial_{t}n_{i}+\tau_{i}^{2}\partial_{t^{2}}n_{i}.$$

In this case the simplest matrix $M_{4}^{\left(3\right)}$ and function $f_{4}^{\left(3\right)}\left(\tau ,\tau_{0}\right)$ have the form:

(87) $$M_{4}^{\left(3\right)}=\left(\begin{array}{cccc} 1 & 0 & 0 & \tau^{3}\\ 0 & \tau_{0}^{2} & 0 & 0\\ 0 & 0 & \tau_{0}^{4} & 0\\ -\tau^{3} & 0 & 0 & \tau_{0}^{6} \end{array}\right),\;f_{4}^{\left(3\right)}\left(\tau ,\tau_{0}\right)=\frac{1}{1+a^{6}},\;a=\frac{\tau }{\tau_{0}}.$$

It is obvious that higher-order equations can also be obtained in the same way. At L/l→0 one observes a decrease in the rates of diffusion and chemical reactions, and the higher-order constitutive equations can be used for description of irreversible processes which occur in nanosystems.

We have, thus, obtained a wider class of constitutive equations compared to the traditional EIT. For the completeness of thermodynamic theory, the following question is very important: whether these constitutive equations can be transformed into the form which is independent of the frame (material-invariant equations)? Several approaches are known for the Maxwell-Cattaneo equation. The most well-known approaches use the Jaumann derivative instead of the usual local time derivative [11]. The modified version of the objective time derivative was used to solve the problem of Marangoni-Bénard instability [34]. In [35,36], the material derivative and the Oldroid’s derivative was used to modify the Maxwell-Cattaneo equation. In this case, it was possible to obtain a single equation for the temperature field [36] and to prove its Galilean invariance. 

However, the question remains: is it possible to apply these approaches to other equations considered in this paper? There is a problem even for the Jeffreys Equation (79) where the term $D_{0i}\eta \nabla \partial_{t}n_{i}$ is added, and is more complex for higher-order Equations (71), (72) and (84). In addition, there is an opinion that frame-indifference is not satisfied in several disciplines [11]. Therefore, there is doubt for its applicability in the general case.

## 9. Discussion and Conclusions 

Thermodynamic theory of nanosystems assumes various approaches, which are based on the introduction of additional variables. One of the first theories is inspired by statistical mechanics, where an ensemble of equivalent systems is considered [37]. In this case, the number of systems is an additional variable. Using this approach, we can generalize the equations of classical thermodynamics and investigate systems at the nanoscale. Unfortunately, transfer processes are not considered in this theory. 

Another approach is based on the introduction of internal variables of nanoparticles (volume or size of particles as additional variables) [38,39]. It is assumed that the system is in the local equilibrium and the well-known formalism of thermodynamics of irreversible processes is used. In this case, stochastic methods based on the Fokker-Planck equation and the Smoluchowski equation can be applied. 

In this paper, we proceeded from classical thermodynamic theory and considered the traditional version of EIT of heat conduction, wherein the extra variables are the higher-order heat fluxes (fluxes of the fluxes). Within this approach the heat transport in the nanosystems and the thermal conductivity as a function of the size of the nanoscale structures were considered [1,2]. 

At the same time, assuming that the entropy density is a function of usual thermodynamic variables and their higher-order time derivatives, we studied the irreversible processes which occur in reaction-diffusion nanosystems. The entropy balance equations were obtained, and the linear phenomenological equations were constructed based on the expression of the entropy production. The characteristic relaxation times, which define the memory effects of irreversible processes connected with the coefficients of phenomenological equations, were introduced, as well. Thus, the diffusion coefficients and the rate constant of the chemical reaction were established as the functions of size of nanosystems, using in the context the linear theory. The proposed thermodynamic theory can be developed in the framework of the linear EIT and proceeds from the constitutive equations, at that the matrix of phenomenological coefficients satisfies the Onsager-Casimir reciprocal relations in the case of the odd number of phenomenological equations. 

Solutions of a linear system of phenomenological equations relative to fluxes and their higher-order time derivatives depend on the matrix of phenomenological coefficients. As in the case of the traditional version of EIT, a series of matrices leads to the solutions which are representable in the form of finite truncations of continued-fraction expansion. Therefore, obtained solutions with increased number of phenomenological equations converge to the finite limit. The established expressions for fluxes and their time derivatives reveal that the rates of diffusion and chemical reactions decreases with reducing dimensions of nanosystems (Equations (63) and (64)). For a constitutive equation with the higher-order time derivatives of flux one can also construct the linear theory, according to which the greater is the dispersion of nanosystems, the lower are the rates of diffusion and chemical reactions.
